# 453. Distribution of cycle threshold values and the associated factors of SARS-CoV-2 omicron variant in a community setting in Japan

**DOI:** 10.1093/ofid/ofad500.523

**Published:** 2023-11-27

**Authors:** Yoshikazu Mutoh, Takumi Umemura, Toshihiko Ichihara

**Affiliations:** Fujita Health University, Seto, Aichi, Japan; Tosei General Hospital, Seto, Aichi, Japan; Tosei General Hospital, Seto, Aichi, Japan

## Abstract

**Background:**

Little is known regarding the viral dynamics of omicron variant, although the number of severe cases of COVID-19 has decreased due to the expansion of vaccines and emerging of omicron variant. Therefore, we investigated the distribution of cycle threshold (Ct) values and related characteristics in this study.

Distribution of cycle threshold values of SARS-Cov-2 PCR by symptoms

**Figure:**
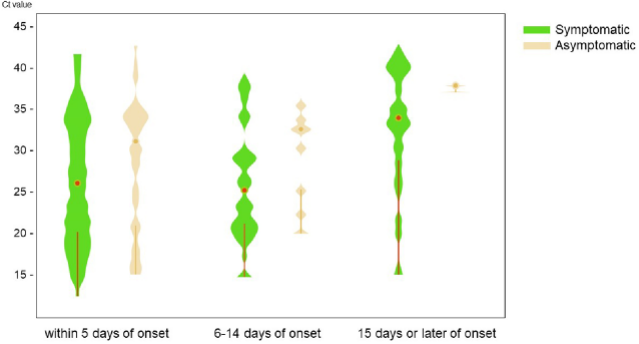

**Methods:**

Patients who tested positive for SARS-CoV-2 using PCR test at Tosei General Hospital between April 2022 and March 2023 were retrospectively analyzed. SARS-CoV-2 was detected using nasopharyngeal swab or saliva via Xpress SARS-CoV-2 assay (Cepheid, Sunnyvale, USA) and Cobas 8800 system (Roche). Age, sex, days since onset, symptoms, comorbidities, prognosis, and vaccination status were collected for each patient. The date of onset was estimated from the contact history of asymptomatic patients.

**Results:**

Of 605 patients, mean age was 51.0 (±26.0) years, 323 were men (53.4 %), 77 (12.7 %) were asymptomatic, and 360 (59.5 %) had more than three times of vaccination. The most frequent comorbidity was chronic pulmonary diseases in 83 (13.7%) patients, followed by diabetes in 61 (10.1%), chronic heart diseases in 61 (10.1%), and malignancy in 55 (9.1%). The distribution of Ct value was 27.8 (IQR: 21.5–34.8) for within 5 days of onset, 27.5 (IQR: 22.5–34.0) for 6–14 days of onset, 35.6 (IQR: 30.4-40.7) for 15 days or later of onset. More than 38.6% (68/176) of the cases diagnosed at symptom onset showed a Ct value of >32.

Among 494 patients diagnosed within 5 days of onset, symptomatic patients showed significantly lower median Ct values than those of not (27.19 vs 32.54; *P value < 0.001*). However, in multivariable analysis, age, disease severity, vaccination status, and comorbidities were not associated with Ct values.

**Conclusion:**

Ct values of SARS-CoV-2 omicron variant might be able to estimate the onset of disease onset. Conversely, certain number of cases revealed high Ct values from the early phase of infection. Physicians should focus on the interpretation of Ct values for infection prevention and control.

**Disclosures:**

**All Authors**: No reported disclosures

